# Synthetic minority over-sampling technique-enhanced machine learning models for predicting recurrence of postoperative chronic subdural hematoma

**DOI:** 10.3389/fneur.2024.1305543

**Published:** 2024-04-22

**Authors:** Zhihui Ni, Yehao Zhu, Yiwei Qian, Xinbo Li, Zhenqiu Xing, Yinan Zhou, Yu Chen, Lijie Huang, Jianjing Yang, Qichuan Zhuge

**Affiliations:** ^1^Department of Neurosurgery, The First Affiliated Hospital of Wenzhou Medical University, Wenzhou, China; ^2^Zhejiang Provincial Key Laboratory of Aging and Neurological Disorder Research, The First Affiliated Hospital of Wenzhou Medical University, Wenzhou, China

**Keywords:** chronic subdural hematoma, machine learning, hematoma recurrence, shapley additive explanation, web calculator

## Abstract

**Objective:**

Chronic subdural hematoma (CSDH) is a neurological condition with high recurrence rates, primarily observed in the elderly population. Although several risk factors have been identified, predicting CSDH recurrence remains a challenge. Given the potential of machine learning (ML) to extract meaningful insights from complex data sets, our study aims to develop and validate ML models capable of accurately predicting postoperative CSDH recurrence.

**Methods:**

Data from 447 CSDH patients treated with consecutive burr-hole irrigations at Wenzhou Medical University’s First Affiliated Hospital (December 2014-April 2019) were studied. 312 patients formed the development cohort, while 135 comprised the test cohort. The Least Absolute Shrinkage and Selection Operator (LASSO) method was employed to select crucial features associated with recurrence. Eight machine learning algorithms were used to construct prediction models for hematoma recurrence, using demographic, laboratory, and radiological features. The Border-line Synthetic Minority Over-sampling Technique (SMOTE) was applied to address data imbalance, and Shapley Additive Explanation (SHAP) analysis was utilized to improve model visualization and interpretability. Model performance was assessed using metrics such as AUROC, sensitivity, specificity, F1 score, calibration plots, and decision curve analysis (DCA).

**Results:**

Our optimized ML models exhibited prediction accuracies ranging from 61.0% to 86.2% for hematoma recurrence in the validation set. Notably, the Random Forest (RF) model surpassed other algorithms, achieving an accuracy of 86.2%. SHAP analysis confirmed these results, highlighting key clinical predictors for CSDH recurrence risk, including age, alanine aminotransferase level, fibrinogen level, thrombin time, and maximum hematoma diameter. The RF model yielded an accuracy of 92.6% with an AUC value of 0.834 in the test dataset.

**Conclusion:**

Our findings underscore the efficacy of machine learning algorithms, notably the integration of the RF model with SMOTE, in forecasting the recurrence of postoperative chronic subdural hematoma. Leveraging the RF model, we devised an online calculator that may serve as a pivotal instrument in tailoring therapeutic strategies and implementing timely preventive interventions for high-risk patients.

## Introduction

Chronic subdural hematoma (CSDH) is a prevalent neurological disorder. Manifesting approximately 3 weeks post-injury, its symptoms encompass focal neurological deficits, cognitive alterations, and signs of elevated intracranial pressure, primarily headaches and reduced consciousness (1). In severe cases, CSDH can be fatal (2). The elderly population, particularly those above 65 years of age, faces heightened risk due to widespread anticoagulation treatments, natural cerebral atrophy, and increased susceptibility to trauma (3, 4). While previously believed to be caused by slow venous bleeding from the brain’s bridging veins following trauma, recent research indicates that CSDH’s onset and recurrence are multifactorial, involving disruption to the cells lining the dura, inflammation, angiogenesis, coagulation disturbances, microbleeds, and exudation (5–7).

Burr-hole irrigation has emerged as an effective treatment for symptomatic CSDH. Yet, despite post-evacuation closed-system drainage, recurrence remains a significant clinical challenge, with rates hovering around 9–33% (7). This recurrence presents considerable clinical conundrums, especially for older patients at elevated risk of neurological and surgical complications (8). Several factors, such as age, previous bleeding episodes, cerebral atrophy, alcohol consumption, the presence of subdural air, radiological signs, and surgical techniques, have been identified as potential contributors to recurrence (9–11). Nevertheless, existing predictive measures have shown inconsistent results and have not seen widespread clinical adoption (12). In recent years, several models predicting the recurrence of CSDH after surgery have been published. To evaluate the performance of these existing models in predicting postoperative recurrence in CSDH patients, Holl et al. (13) utilized a retrospective database comprising data from 2,384 patients across three regions in the Netherlands. The study revealed that current predictive models perform poorly on the author’s dataset, failing to effectively forecast the recurrence of hematomas following CSDH treatment. This research highlights the challenges of predicting hematoma recurrence after CSDH treatment and underscores the necessity of adopting appropriate modeling strategies to develop high-quality models.

Machine learning (ML) has gained increasing influence in medical research, offering the ability to uncover hidden patterns and correlations from vast datasets (14). Its capability for identifying complex data associations, often overlooked by traditional statistical methods, provides invaluable insights for both clinicians and researchers (15). Machine learning models hold significant potential in neurosurgical predictive analytics and have been extensively applied to forecast acute hematoma expansion in cerebral hemorrhage, predict meningioma grade, and prognosticate outcomes for glioma patients, among other uses (16–18). These models empower physicians and patients to make more informed decisions and offer personalized medical services (19). SMOTE is an approach designed to tackle the issue of data imbalance, particularly within the fields of machine learning and statistics. It operates by inserting new synthetic samples between minority class instances, thus oversampling the dataset to increase the number of minority class samples. While this method effectively mitigates data imbalance, it may lack precision when dealing with samples near the decision boundary. The core concept of border-line SMOTE is to concentrate on those minority class samples that are challenging to classify, specifically those located near the boundary between majority and minority classes. These samples are deemed crucial for constructing the decision boundary. Therefore, generating more synthetic samples around these boundary-line samples can aid classifiers in better learning the characteristics of these complex regions, thereby enhancing the classification performance for minority class samples (20). Considering this, our study aims to develop and assess ML models, supplemented with SMOTE, for the accurate prediction of postoperative CSDH recurrence.

## Methods

### Study population

**Figure 1:**
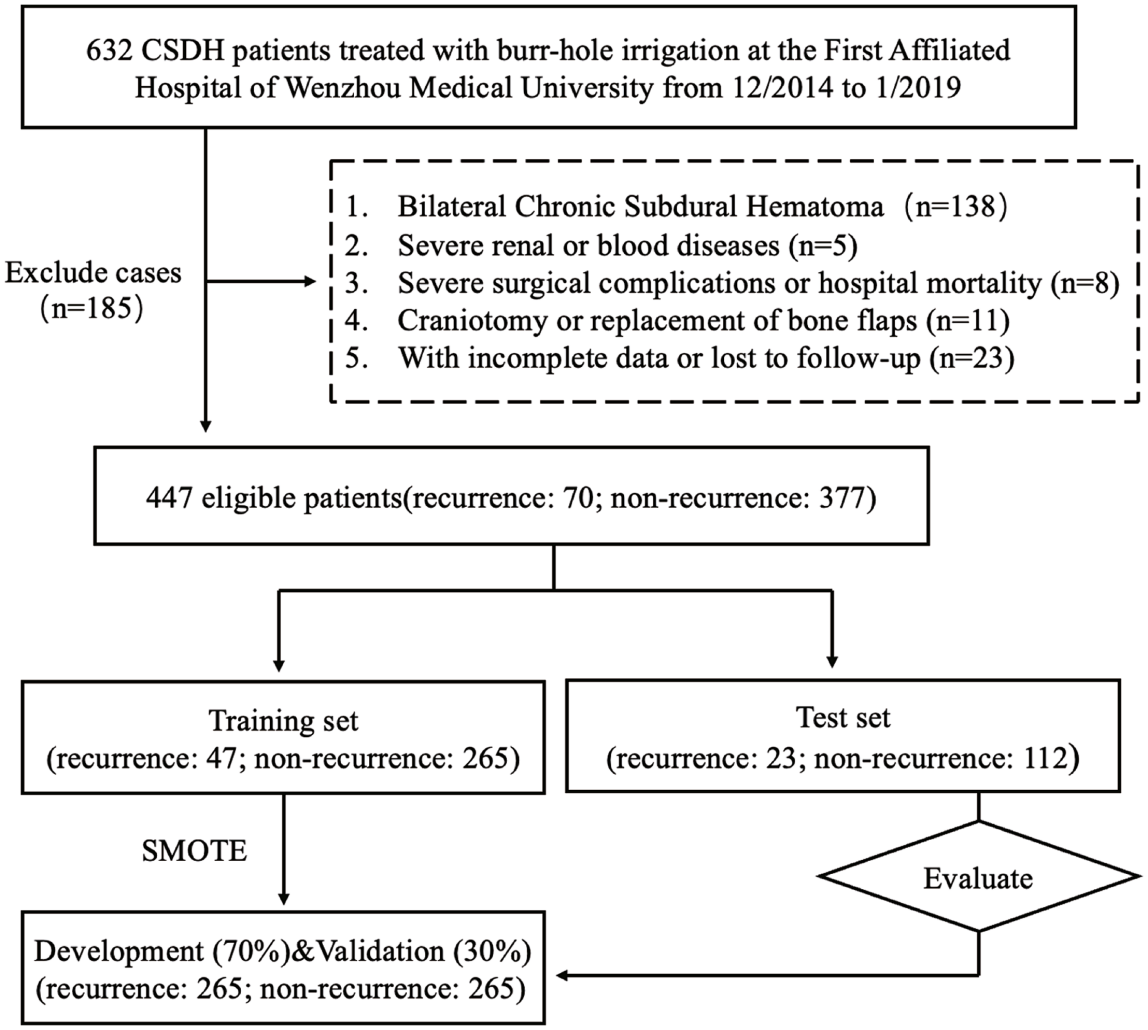
Flowchart of the study procedure. CSDH, chronic subdural hematoma; SMOTE: synthetic minority over-sampling technique.

We conducted a retrospective analysis of medical records from patients diagnosed with CSDH who underwent surgical treatment at the Department of Neurosurgery, First Affiliated Hospital of Wenzhou Medical University, from December 2014 to January 2019. Diagnoses of CSDH were confirmed via head MRI and CT scans. This study received approval from the Committee for Ethics in Clinical Research, and due to its retrospective design, there was no need for informed patient consent. Out of 632 adults diagnosed with CSDH, 447 with unilateral CSDH who underwent burr-hole irrigation were included in the final analysis. We excluded patients with: (1) bilateral chronic subdural hematoma, (2) severe renal or hematological conditions, (3) significant surgical complications or in-hospital fatalities, (4) those who underwent craniotomy or bone flap replacements, and (5) cases with incomplete records or lost during follow-up (Figure 1).

### Surgical procedures and management

Under general anesthesia, all patients underwent the standard burr-hole irrigation (BHI) procedure. A single burr hole was strategically placed at the hematoma’s thickest region, followed by saline irrigation. Subsequently, a silicone catheter equipped with a closed subdural drainage system was positioned within the hematoma cavity. Typically, the catheter was extracted between 24 to 72 h postoperatively, contingent upon drainage volume. Postoperative administration of atorvastatin, sustained for a minimum of 1 month, facilitated the absorption of residual hematoma and minimized the risk of recurrence (this approach is grounded on the findings of a randomized clinical trial conducted by Jiang et al. (21), which demonstrated the safety and efficacy of atorvastatin in promoting hematoma absorption in Chinese patients with CSDH).

### Model input features selected

**Figure 2:**
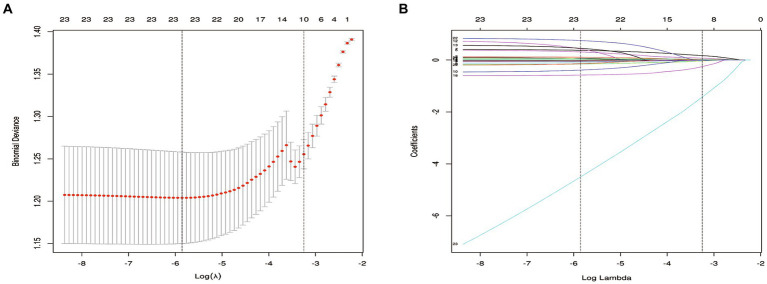
Key feature selection using the LASSO regression.

We collected data from patient records, encompassing 32 unique clinical, radiological, and laboratory test characteristics. These included demographic features (age, gender) and lifestyle behaviors such as smoking and alcohol consumption. Pertinent comorbidities captured were hypertension, diabetes, and heart disease, along with any history of cranial trauma. Blood pressure measurements, including systolic (SBP) and diastolic (DBP), were documented. Initial CT or MRI scans, reviewed independently by two authors (LH and XL), revealed the hematoma’s location and maximum diameter. Admission laboratory metrics comprised white blood cell count (WBC), neutrophil percentage and count (NEUT), lymphocyte count (LYM), red blood cell count (RBC), hemoglobin (HB), platelet count (PLT), prothrombin time (PT), international normalized ratio (INR), fibrinogen (FIB), activated partial thromboplastin time (APTT), thrombin time (TT), total bilirubin (TB), direct bilirubin (DB), indirect bilirubin (IB), albumin (ALB), alanine aminotransferase (ALT), aspartate aminotransferase (AST), blood glucose (GLU), urea nitrogen (UN), and creatinine (CR). Using the least absolute shrinkage and selection operator (LASSO) regression technique, we identified 10 features significantly correlated with CSDH recurrence (Figure 2).

### Assessment of CSDH recurrence

Patients underwent a head CT or MRI scan within the initial 48 h following surgery, and again on days 6 or 7 post-operation. To ensure comprehensive evaluation, a subsequent CT scan or MRI was performed at the outpatient clinic 3 months post-surgery. The recurrence of CSDH was determined using specific radiological criteria. Regardless of any subsequent surgical interventions, any observed increase in subdural fluid volume and concurrent brain compression in either hemisphere, within 3 months post-surgery relative to the initial postoperative CT, was deemed a recurrence. This determination was collaboratively made by two seasoned neurosurgeons, uninformed of the study’s particulars.

### Balancing data for enhanced predictive modeling

**Figure 3:**
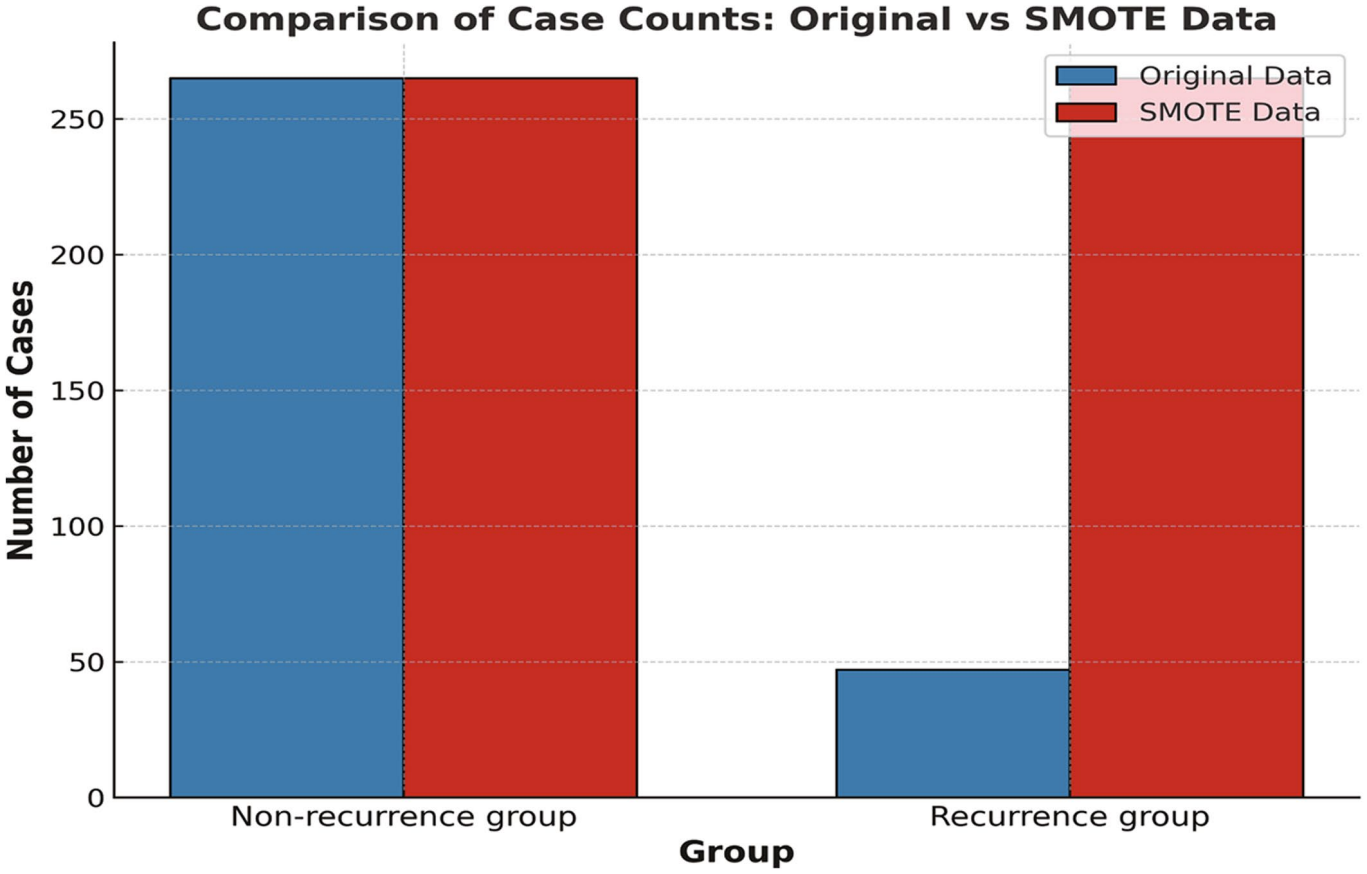
Comparison of case counts: Original vs SMOTE Data.

Data from 312 out of 447 CSDH patients were utilized for the construction of the model, while the remaining 135 patients’ data were employed to assess the model’s predictive performance. In the training subset (312 patients), CSDH recurrence occurred in 15.1% (47 patients), whereas the remaining 84.9% (265 patients) experienced no recurrence. This significant data imbalance could compromise the performance of the predictive model. Balanced datasets are known to improve predictive accuracy. To address this issue, we employed the borderline-SMOTE, an advanced oversampling technique commonly used in machine learning. This approach improves upon the conventional SMOTE by targeting minority class samples located at the boundary between the majority and minority classes—areas where misclassification is prone to occur. By generating new samples around these crucial instances, Borderline-SMOTE enhances the classifier’s discriminatory ability, particularly when class overlap is present. The application of borderline-SMOTE increased the minority class representation in our training dataset from 15.1 to 50%. This resulted in equal representation of CSDH patient groups, each with 265 instances of recurrence and non-recurrence (shown in Figure 3). This expanded dataset of 530 data points was subsequently divided into development (70%) and validation (30%) subsets for machine learning model construction.

### Statistical analysis

Statistical analyses were performed using R statistical software (version 3.6.3, https://www.r-project.org/) and Python software (version 3.7, https://www.python.org/). The Shapiro–Wilk test was employed to assess the distribution of continuous variables. Continuous variables with a Gaussian distribution were expressed as mean ± SD and subjected to an independent-sample *t*-test. Non-normally distributed variables were represented as median with interquartile range (IQR) and analyzed using the Mann–Whitney *U* test. Categorical variables were presented as frequencies and percentages and analyzed with either the chi-square test or Fisher’s exact test, as appropriate.

For optimal predictive performance, we constructed eight models: extreme gradient boosting (XGBoost), logistic regression (LR), light gradient boosting machine (LGBM), random forest (RF), adaptive boosting (AdaBoost), multi-layer perceptron (MLP), support vector machine (SVM), and Gaussian Naive Bayes (GNB). These models were built upon features selected through LASSO regression. Validation sets were used to evaluate the performance of each classifier, measuring the area under the receiver operating characteristic curve (AUROC). Sensitivity, specificity, F1 score, and overall accuracy were also assessed for each algorithm. The model with the highest AUC was deemed superior. Shapley additive explanation (SHAP) analysis further clarified the importance of each feature, enhancing the visualization and interpretability of the model.

## Results

### Demographic and clinical features

We incorporated a cohort of 447 individuals into our study. Within 3 months post-surgery, 70 patients, representing 15.6% of the sample, experienced a recurrence of chronic subdural hematoma (CSDH). Intriguingly, the mean age of patients in the recurrence group was 73.51 ± 10.74 years, significantly higher than that of the non-recurrence group at 69.60 ± 12.20 years. Moreover, at admission, the recurrence group had a median platelet count of 194 [161, 232], which was notably lower than the 213 [170, 252] observed in the non-recurrence group. Further analysis indicated an increased susceptibility to hematoma recurrence in patients with lower fibrinogen concentrations or elevated urea nitrogen levels. Comprehensive clinical data is available in Table 1. For robust analysis, the CSDH patient cohort was divided into training and validation subsets, adhering to a 7:3 ratio. Importantly, there were no significant differences in demographic or clinical characteristics between the training and test groups (Table 2).

**Table 1:** Comparison variables between non-recurrence group and recurrence group.

Characteristic	Total (*n* = 447)	Non-recurrence (*n* = 377)	Recurrence (*n* = 70)	*p*-value
Gender, *n*%				0.586
Female	67 (14.98)	58 (15.38)	9 (12.85)	
Male	38 (85.01)	319 (84.61)	61 (87.14)	
Age, median (*Q*1, *Q*3)	70.21 ± 12.0	69.60 ± 12.20	73.51 ± 10.74	0.008
Hypertension, *n*%	173 (38.70)	149 (39.52)	24 (34.28)	0.409
Diabetes, *n*%	53 (11.85)	45 (11.93)	8 (11.42)	0.904
Smoking, *n*%	173 (38.70)	151 (40.05)	22 (31.42)	0.174
Drinking, *n*%	163 (36.46)	136 (36.07)	27 (38.571)	0.690
SBP, mean (SD)	140.08 ± 19.96	140.59 ± 19.87	137.34 ± 20.18	0.212
DBP, mean (SD)	79.45 ± 11.35	79.89 ± 11.52	77.11 ± 10.05	0.060
Trauma history, *n*%	297 (66.443)	255 (67.639)	42 (60)	0.214
Heart disease, *n*%	30 (6.711)	28 (7.427)	2 (2.857)	0.161
Hematoma location, *n*%				0.732
Left	251 (56.15)	213 (56.49)	38 (54.28)	
Right	196 (43.84)	164 (43.50)	32 (45.71)	
HWD, median (*Q*1, *Q*3)	22 [18, 26]	22 [18, 26]	22 [18, 25]	0.931
WBC, median (*Q*1, *Q*3)	6.80 [5.73, 8.21]	6.87 [5.73, 8.43]	6.50 [5.71, 7.55]	0.086
NEUT median (*Q*1, *Q*3)	4.50 [3.59, 5.93]	4.56 [3.58, 6.06]	4.30 [3.70, 5.10]	0.177
Lymphocyte, median (*Q*1, *Q*3)	1.49 [1.14, 1.82]	1.50 [1.14, 1.83]	1.46 [1.10, 1.79]	0.852
NEUT percentage, mean (SD)	0.675 ± 0.107	0.675 ± 0.110	0.672 ± 0.091	0.808
RBC, median (*Q*1, *Q*3)	4.28 [3.92, 4.64]	4.28 [3.91, 4.67]	4.23 [3.99, 4.50]	0.528
HB, median (*Q*1, *Q*3)	133 [121, 143]	133 [121, 144]	132 [122, 139]	0.290
PLT, median (*Q*1, *Q*3)	207 [169, 249]	213 [170, 252]	194 [161, 232]	0.034
PT, median (*Q*1, *Q*3)	13.4 [12.8, 13.9]	13.4 [12.9, 13.9]	13.3 [12.8, 14.0]	0.799
INR, median (*Q*1, *Q*3)	1.03 [0.97, 1.08]	1.030 [0.98, 1.08]	1.02 [0.97, 1.09]	0.775
Fibrinogen, median (*Q*1, *Q*3)	3.61 [3.10, 4.24]	3.63 [3.15, 4.27]	3.44 [2.90, 3.98]	0.049
APTT ratio, median (*Q*1, *Q*3)	1.00 [0.92, 1.08]	1.00 [0.92, 1.09]	0.99 [0.92, 1.06]	0.419
TT, median (*Q*1, *Q*3)	16.0 [15.5, 16.8]	16.0 [15.4, 16.8]	16.1 [15.5, 16.9]	0.379
GLU, median (*Q*1, *Q*3)	5.5 [4.7, 6.5]	5.4 [4.7, 6.5]	5.6 [5.0, 6.6]	0.406
UN, median (*Q*1, *Q*3)	4.9 [4.0, 6.3]	4.8 [3.9, 6.1]	5.7 [4.3, 6.9]	0.006
CR, median (*Q*1, *Q*3)	67 [58, 77]	67 [58, 77]	67 [60, 78]	0.290
ALT, median (*Q*1, *Q*3)	16 [11, 23]	16 [11, 23]	16 [11, 22]	0.657
AST, median (*Q*1, *Q*3)	21 [17, 26]	21 [17, 26]	20 [18, 25]	0.897
TB, median (*Q*1, *Q*3)	10 [8, 14]	10 [8, 14]	12 [9, 15]	0.188
DB, median (*Q*1, *Q*3)	4 [3, 6]	4 [3, 5]	5 [3, 6]	0.203
IB, median (*Q*1, *Q*3)	6 [5, 9]	6 [4, 9]	6 [5, 9]	0.397

SBP, systolic blood pressure; DBP, diastolic blood pressure; HWD, hematoma widest diameter; WBC, white blood cell; NEUT, neutrophil; RBC, red blood cell; HB, hemoglobin; PLT, platelets; PT, prothrombin time; INR, international normalized ratio; APTT, activated partial thromboplastin time; TT, thrombin time; GLU, blood glucose; UN, urea nitrogen; CR, creatinine; ALT, alanine aminotransferase; AST, aspartate aminotransferase; TB, total bilirubin; DB, direct bilirubin; IB, indirect bilirubin.

**Table 2:** Clinical characteristics of patients.

Characteristic	Total (*n* = 447)	Training set (*n* = 312)	Test set (*n* = 135)	*p*-value
Gender, *n*%				0.425
Female	67 (14.98)	44 (14.10)	23 (17.03)	
Male	380 (85.01)	268 (85.89)	112 (82.96)	
Age, median (*Q*1, *Q*3)	71 [63, 79]	72 [64, 80]	69 [63, 79]	0.076
Hypertension, *n*%	173 (38.70)	120 (38.46)	53 (39.25)	0.874
Diabetes, *n*%	53 (11.85)	36 (11.53)	17 (12.59)	0.752
Smoking, *n*%	173 (38.70)	116 (37.17)	57 (42.22)	0.315
Drinking, *n*%	163 (36.465)	117 (37.500)	46 (34.074)	0.490
SBP, mean (SD)	140.08 ± 19.96	140.40 ± 19.94	139.34 ± 19.98	0.610
DBP, mean (SD)	79.45 ± 11.35	79.44 ± 11.46	79.48 ± 11.07	0.978
Trauma history, *n*%	297 (66.44)	208 (66.66)	89 (65.92)	0.879
Heart disease, *n*%	30 (6.71)	17 (5.44)	13 (9.63)	0.105
Hematoma location, *n*%				0.804
Left	251 (56.15)	174 (55.76)	77 (57.03)	
Right	196 (43.84)	138 (44.23)	58 (42.96)	
HWD, median (*Q*1, *Q*3)	22 [18, 26]	22 [18, 26]	23 [18, 25]	0.910
WBC, median (*Q*1, *Q*3)	6.80 [5.73, 8.21]	7.00 [5.74, 8.42]	6.55 [5.71, 7.69]	0.102
Neutrophil, median (*Q*1, *Q*3)	4.50 [3.59, 5.93]	4.73 [3.60, 6.06]	4.30 [3.58, 5.23]	0.059
Lymphocyte, median (*Q*1, *Q*3)	1.49 [1.14, 1.82]	1.48 [1.10, 1.83]	1.51 [1.20, 1.80]	0.588
Neutrophil percentage, mean (SD)	0.675 ± 0.107	0.679 ± 0.107	0.664 ± 0.107	0.155
RBC, median (*Q*1, *Q*3)	4.28 [3.92, 4.64]	4.28 [3.90, 4.63]	4.28 [3.99, 4.64]	0.687
HB, median (*Q*1, *Q*3)	133 [121, 143]	133 [120, 144]	132 [123, 142]	0.804
PLT, median (*Q*1, *Q*3)	207 [169, 249]	210 [169, 250]	203 [168, 245]	0.827
PT, median (*Q*1, *Q*3)	13.4 [12.8, 13.9]	13.3 [12.8, 13.9]	13.4 [12.9, 13.9]	0.643
INR, median (*Q*1, *Q*3)	1.03 [0.97, 1.08]	1.02 [0.98, 1.08]	1.04 [0.97, 1.08]	0.858
Fibrinogen, median (*Q*1, *Q*3)	3.61 [3.10, 4.24]	3.59 [3.10, 4.15]	3.63 [3.08, 4.29]	0.864
APTT ratio, median (*Q*1, *Q*3)	1 [0.92, 1.08]	1.010 [0.92, 1.08]	0.990 [0.92, 1.09]	0.766
TT, median (*Q*1, *Q*3)	16.0 [15.5, 16.8]	16.0 [15.4, 16.7]	16.1 [15.6, 16.9]	0.067
GLU, median (*Q*1, *Q*3)	5.5 [4.7, 6.5]	5.4 [4.7, 6.5]	5.5 [4.7, 6.6]	0.861
UN, median (*Q*1, *Q*3)	4.9 [4.0, 6.3]	4.9 [4.0, 6.3]	4.8 [4.1, 5.9]	0.345
CR, median (*Q*1, *Q*3)	67 [58, 77]	67 [58, 78]	67 [58, 76]	0.854
ALT, median (*Q*1, *Q*3)	16 [11, 23]	16 [11, 22]	17 [12, 23]	0.397
AST, median (*Q*1, *Q*3)	21 [17, 26]	20 [17, 26]	21 [18, 24]	0.857
Total bilirubin, median (*Q*1, *Q*3)	10 [8, 14]	11 [8, 14]	10 [8, 14]	0.713
Direct bilirubin, median (*Q*1, *Q*3)	4 [3, 6]	4 [3, 5.8]	4 [3, 6]	0.814
Indirect bilirubin, median (*Q*1, *Q*3)	6 [5, 9]	6 [4, 8.6]	6 [5, 9]	0.631

SBP, systolic blood pressure; DBP, diastolic blood pressure; HWD, hematoma widest diameter; WBC, white blood cell; RBC, red blood cell; HB, hemoglobin; PLT, platelets; PT, prothrombin time; INR, international normalized ratio; APTT, activated partial thromboplastin time; TT, thrombin time; GLU, blood glucose; UN, urea nitrogen; CR, creatinine; ALT, alanine aminotransferase; AST, aspartate aminotransferase.

### Key variables

Figures 3A,B presents the 10 features with nonzero coefficients determined via LASSO regression analysis, utilizing 10-fold cross-validation to ascertain the optimal lambda value. The following 10 factors, significantly associated with CSDH recurrence, comprise urea nitrogen (UN), aspartate aminotransferase (AST), direct bilirubin (DB), thrombin time (TT), fibrinogen (FIB), systolic blood pressure (SBP), hematoma’s widest diameter (HWD), diabetes, age, and a history of heart diseases.

### Model performance

**Figure 4:**
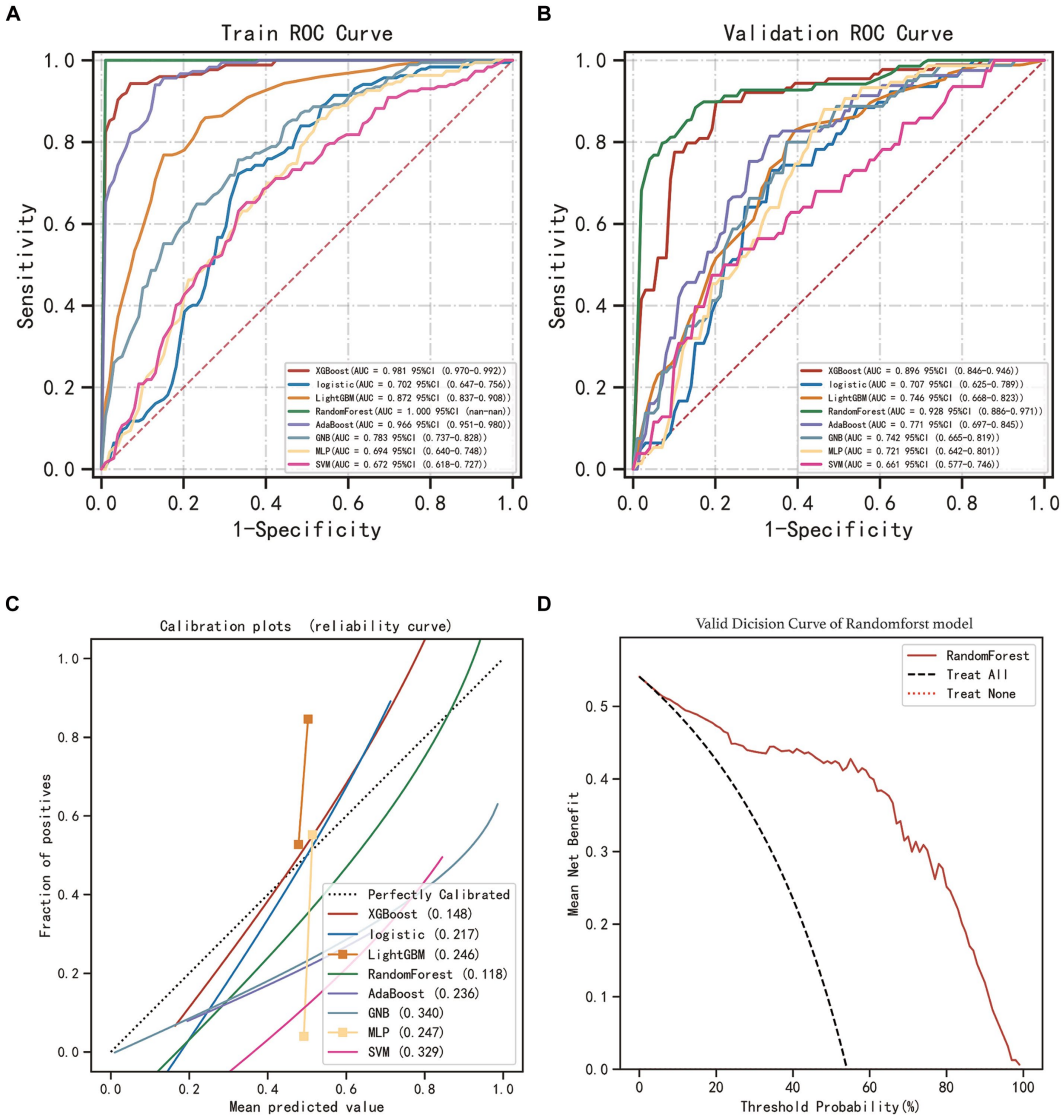
Performance assessment of the models. **(A)** Receiver operating characteristic curve (ROC) of eight machine learning models in training set. **(B)** ROC of models in validation set. **(C)** Calibration plots of models in the validation set. **(D)** Decision curve analysis (DCA) for RF model in the validation set.

After identifying the 10 key variables, we utilized machine learning algorithms to predict CSDH recurrence. The predictive accuracy of these models was evaluated using essential metrics, including AUC, precision, recall, specificity, and F1 score. The results are provided in Table 3. The random forest (RF) model outperformed the others in the validation set, as evidenced by an AUC value of 0.928 from the ROC curve. Figure 4 compares the ROC curves of the eight models and displays their calibration plots. It also shows the decision curve analysis of the random forest model, indicating its net clinical benefit compared to a universal treatment strategy.

**Table 3:** Performance of models in the validation set.

Model	AUC	Accuracy	Sensitivity	Specificity	F1 score
XGBoost	0.896	0.824	0.899	0.800	0.892
LR	0.707	0.698	0.731	0.667	0.704
LightGBM	0.746	0.686	0.830	0.606	0.778
RandomForest	0.928	0.862	0.884	0.856	0.873
AdaBoost	0.771	0.723	0.815	0.667	0.748
GNB	0.742	0.698	0.800	0.633	0.734
MLP	0.721	0.692	0.880	0.536	0.731
SVM	0.661	0.610	0.474	0.815	0.526

AUC, area under the receiver operating characteristic; XGBoost, extreme gradient boosting; LR, logistic regression; LightGBM, light gradient boosting machine; RF, random forest; AdaBoost, adaptive boosting; GNB, Gaussian NB; MLP, multilayer perceptron; SVM, support vector machine.

### Relative importance of variables in RF model

**Figure 5:**
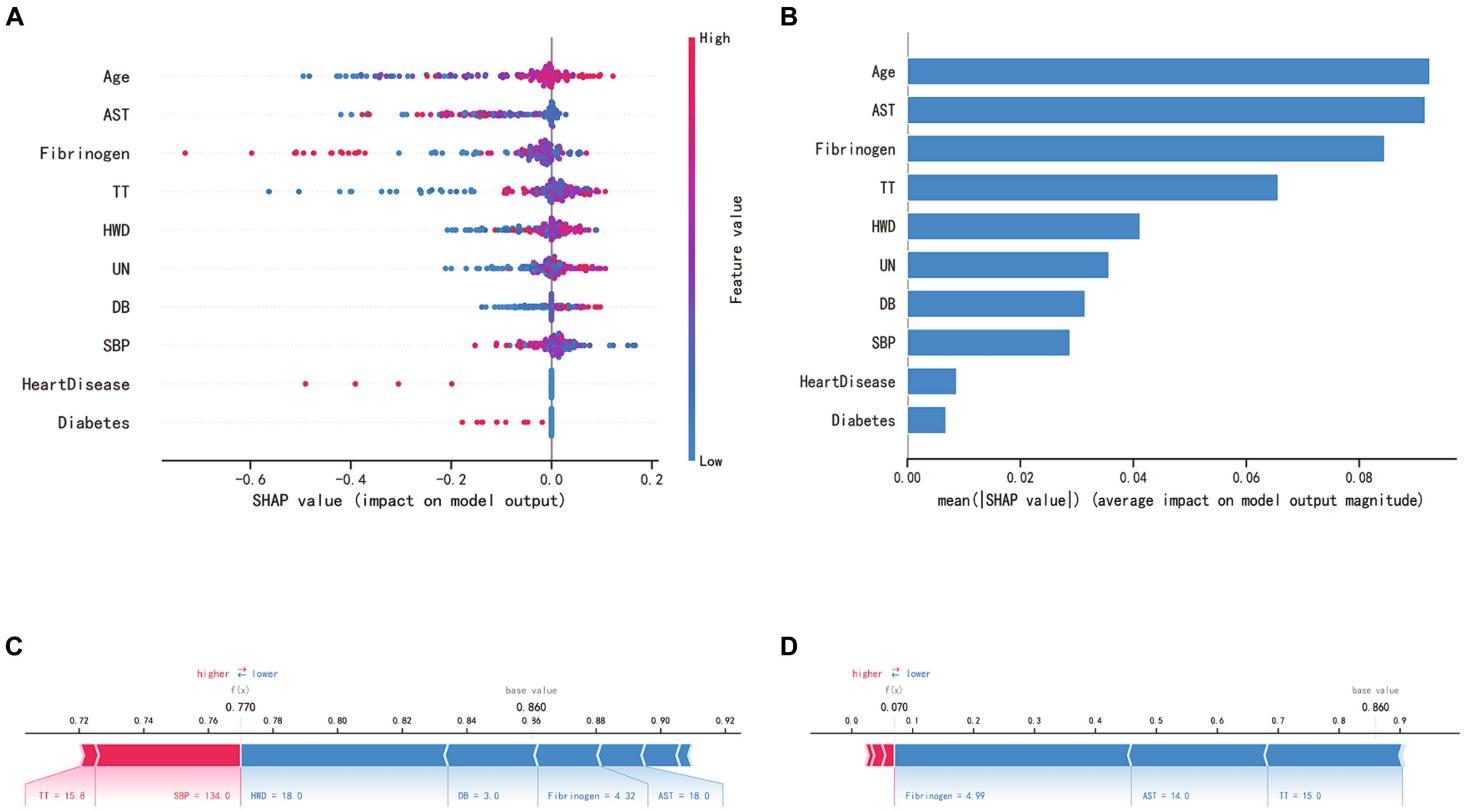
SHAP interprets the RF model. **(A)** SHAP analysis was conducted on the RF model, and the graph depicts each variable as a row with the horizontal axis representing its SHAP value, which indicates the impact of the variable on the risk of CSDH recurrence. Each point corresponds to a patient, with red denoting a higher value and blue a lower value. **(B)** The significance of each variable in the RF model is evaluated by computing the average of the absolute SHAP values associated with that variable. **(C**, **D)** The contributing variables are presented in a horizontal sequence, arranged according to the absolute magnitude of their impact. The output value denotes the predicted risk of CSDH recurrence. **(C)** showcases a patient predicted to have a high risk of hematoma recurrence, whereas **(D)** depicts a patient with a predicted low risk of recurrence.

SHAP analysis enabled an unbiased interpretation of the features. In the random forest (RF) model, the clinically significant variables were ranked as follows: age, aspartate aminotransferase (AST), fibrinogen (FIB), thrombin time (TT), hematoma’s widest diameter (HWD), urea nitrogen (UN), direct bilirubin (DB), systolic blood pressure (SBP), and the presence of heart diseases and diabetes in medical history. The SHAP value of a feature directly corresponds to the likelihood of CSDH recurrence. High feature values are represented in red, average values in purple, and low values in blue (see Figures 5A,B). Figure 5C displays the forecasted recurrence probability for a high-risk CSDH patient, while Figure 5D shows the forecast for a low-risk CSDH patient.

### Performance evaluation of RF model using the test set

**Figure 6:**
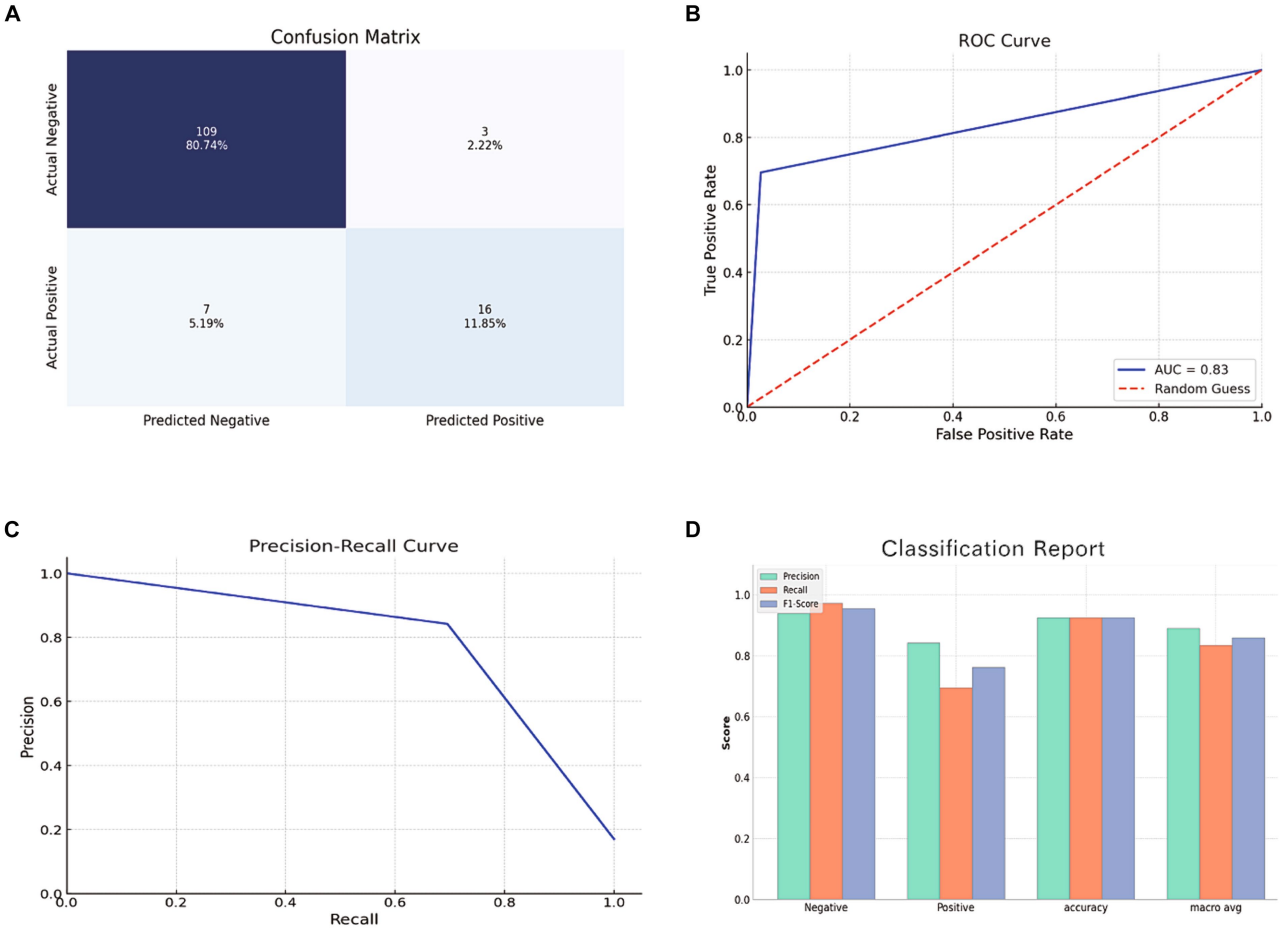
Evaluation of RF model using the test set. **(A)** Confusion Matrix showing the number of true positives, true negatives, false positives, and false negatives. **(B)** ROC Curve depicting the true positive rate against the false positive rate. **(C)** Precision-Recall Curve showing the relationship between precision and recall for different thresholds. **(D)** Bar chart representing the precision, recall, and F1 score for both positive and negative classes.

The RF model, tested on a set of 135 samples, demonstrated an impressive accuracy of 92.6%, precision of 84.2%, recall of 69.6%, an F1 score of 76.2%, and an AUC of 83.4%. Figure 6 depicts a bar chart providing a comprehensive visual overview of the model’s predictive capabilities, including the ROC curve, confusion matrix, precision-recall curve, and a classification report.

### Web-based calculator

**Figure 7:**
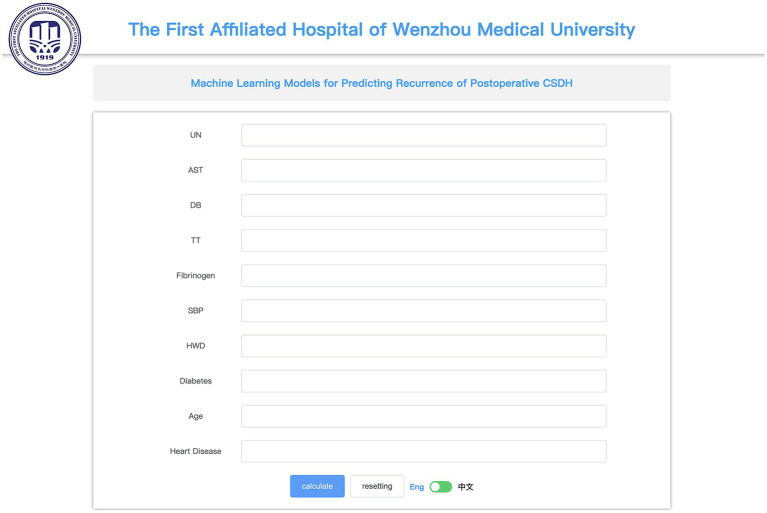
An online calculator constructed with the RF (Random Forest) model.

Utilizing the RF model, we designed an online calculator available at^1^ (Figure 7).

## Discussion

Postoperative recurrence of chronic subdural hematoma (CSDH) is a common challenge in neurology, particularly after interventions like burr-hole irrigation. The high recurrence rate complicates clinical management due to associated neurological impairments and potential increased mortality risks (22, 23). However, there are few precise models for predicting post-surgical CSDH recurrence. To address this gap, we used machine learning to predict CSDH recurrence postoperatively, demonstrating that the random forest (RF) algorithm was particularly accurate.

In this study, we utilized data from 312 patients to construct our model, while the remaining 135 patients’ data were employed to assess the model’s predictive performance. Despite the relatively limited sample size, we ensured the accuracy and reliability of our machine learning (ML) model through careful selection of the model, optimization of evaluation methods, and stringent overfitting control measures. Specifically, we chose the random forest (RF) model for its efficiency in handling the complexity of the dataset and its ability to minimize the risk of overfitting. The RF model is renowned for its robustness and its capacity to provide accurate predictions even in smaller datasets, thanks to its ensemble learning approach, which enhances prediction accuracy by combining multiple decision trees to effectively control overfitting. By implementing rigorous cross-validation techniques and appropriate overfitting prevention measures, we further ensured the optimization of model performance. These meticulously designed steps enabled the RF model to achieve an accuracy rate of 92.6% and an area under the curve (AUC) value of 0.834 in the test dataset, thereby highlighting the model’s high reliability and its capability to precisely predict recurrence risk.

Given the prediction of chronic subdural hematoma (CSDH) recurrence involves interpreting patients’ demographic, laboratory, and radiological characteristics, the complexity of these datasets necessitates the use of a variety of algorithms to capture different information and relationships within the data. Each model has its unique method of processing data and learning patterns, thereby, a multi-model strategy enhances the opportunity to capture all potential correlations within the data. This study adopted a comprehensive strategy to construct eight commonly used machine learning predictive models, aimed at assessing and utilizing the complex dataset from multiple perspectives to identify the most suitable model for predicting postoperative recurrence of CSDH. Through this approach, our research not only demonstrates the potential of machine learning in medical prediction but also provides a practical tool to help improve treatment outcomes for CSDH patients.

To enhance our machine learning model’s accuracy in identifying patients at high risk of CSDH recurrence, we utilized the synthetic minority over-sampling technique (SMOTE) to counter imbalanced data. In many clinical datasets, an unequal distribution of instances between classes can impair machine learning model performance. Often, the model may disproportionately favor the majority class, potentially resulting in suboptimal performance for the minority class (24). The border-line SMOTE method tackles this by generating synthetic samples for the underrepresented class, balancing the dataset (25). Our study suggests that machine learning algorithms, specifically the RF algorithm, can accurately predict and assist healthcare professionals in identifying patients at a high risk of CSDH recurrence.

For feature selection, we employed LASSO regression, suitable for datasets with a high feature-to-observation ratio. LASSO penalizes the absolute values of coefficients, causing some of the less relevant feature coefficients to shrink to zero and effectively removing them from the model (26). This allows the model to focus on genuinely significant predictive factors, improving both the comprehensibility and accuracy of predictions. Given its exceptional performance in our study, we selected the random forest model. During training, random forest builds multiple decision trees and generates classification patterns. The diverse decision trees, derived from various subsets of data and features, enhance the RF model’s ability to prevent overfitting and capture complex feature interactions, offering a more detailed insight into the factors contributing to CSDH recurrence.

We incorporated SHAP analysis into our predictive model, enhancing its interpretability. Transparency is vital in the medical field for understanding the reasoning behind predictions, gaining clinicians’ trust, and encouraging practical application (27). SHAP values provide this transparency by illuminating the individual contributions of each predictive factor. SHAP values elucidate the extent to which each feature influences the model’s prediction outcomes. Positive SHAP values indicate that the presence or increase of a feature tends to elevate the value of the model’s target variable (e.g., the likelihood of CSDH recurrence), signifying it as a risk factor. Conversely, negative SHAP values suggest that the presence or increase of a feature tends to decrease the model’s target variable value, marking it as a protective factor. In Figure 5A, we observed that older age, prolonged prothrombin time, thicker maximum hematoma width, elevated urea nitrogen (UN) levels, and higher direct bilirubin were identified as risk factors. On the other hand, heart disease, diabetes, elevated levels of aspartate aminotransferase, higher fibrinogen, and increased systolic blood pressure (SBP) were associated with protective factors. However, it is important to note that in the univariate analysis between the recurrence and non-recurrence groups, only differences in age, fibrinogen, and urea nitrogen were statistically significant (Table 1). Therefore, for assessing the risk of hematoma recurrence in patients with chronic subdural hematoma, we recommend inputting all factors into our model for evaluation, rather than relying on any single factor to determine the risk of recurrence.

In our RF model’s SHAP analysis, age emerged as the most significant predictive factor for CSDH recurrence, consistent with prior research indicating higher recurrence rates in older patients (28). Several factors may contribute to this pattern. First, brain atrophy could expand the subdural space, facilitating hematoma formation. Second, older patients are more likely to use anticoagulant medications, potentially increasing bleeding tendencies (23). Finally, an increased susceptibility to injury may result in more frequent subdural hematomas. Apart from age, our model highlighted other factors associated with CSDH recurrence, emphasizing the condition’s multifactorial nature (29). AST levels may indicate liver or muscle damage, suggesting systemic inflammation or coagulation disorders that increase hematoma formation and recurrence risk (30). Fibrinogen, a key clotting factor, may be linked to clotting disorders or hypercoagulability affecting CSDH recurrence (31). Abnormal TT could signal coagulation disorders, predisposing individuals to recurrent bleeding (32). HWD directly reflects hematoma size and influences recurrence. Larger hematomas might require more extensive surgical intervention, creating a larger post-drainage hematoma cavity and raising recurrence risk (33). Elevated UN levels may indicate kidney dysfunction or dehydration, potentially affecting blood viscosity, coagulation, and CSDH recurrence (34). High DB levels might correlate with liver disease, affecting clotting factors and increasing recurrence risk (34). Hypertension is known to increase the risk of rebleeding (35). However, in our SHAP analysis, higher systolic blood pressure (SBP) exhibited protective tendencies, albeit without statistical significance. We hypothesize that in patients with chronic subdural hematoma (CSDH), elevated blood pressure might help maintain intracranial pressure, preventing the reaccumulation of blood. Alternatively, the CSDH patient population with higher SBP might exhibit differences in characteristics not thoroughly examined, which could be related to the risk of recurrence. Nonetheless, it is important to underscore that these interpretations require validation through further research. The role of blood pressure management in CSDH is complex; high SBP may interact with various factors, influencing the risk of CSDH recurrence. Furthermore, hypertension itself is a significant risk factor for several cardiovascular diseases, and maintaining high SBP is not recommended in the long term. Therefore, caution should be exercised in interpreting this association, to avoid viewing high SBP as a protective factor while neglecting proper management of hypertension.

In our study, patients with heart disease and diabetes exhibited a tendency towards lower recurrence rates of chronic subdural hematoma (CSDH), although the difference was not statistically significant. This observation contradicts our prior understanding (23, 36). We speculate that one possible explanation for this phenomenon could be the long-term medication regimen these patients often require, which includes antihypertensives, glycemic control medications, and anticoagulant or antiplatelet drugs. It is conceivable that the initial CSDH in these patients may be associated with their medication use. Post-surgical treatment might then receive more personalized and cautious management, potentially reducing the likelihood of CSDH recurrence. Alternatively, the observed phenomenon in the study could be partially attributed to selection bias, suggesting that patients with heart disease and diabetes might receive more aggressive management and treatment for reasons not fully identified, which could indirectly lower their risk of CSDH recurrence.

Prior research has indicated that patients who take oral anticoagulants face a heightened risk of recurrent subdural hematoma (SDH) bleeding and exhibit larger hematoma volumes. Additionally, the use of these medications is associated with the chronic progression of SDH and an increased mortality rate (37). On the other hand, a study found that antiplatelet drug use is related to a higher risk of postoperative rebleeding, whereas anticoagulant use does not show a similar correlation (38). There is also evidence suggesting that early surgical intervention and the immediate resumption of antiplatelet medication after surgery can decrease the risk of thromboembolic complications without increasing the risk of chronic subdural hematoma recurrence. Moreover, extending the period of medication cessation before surgery does not offer significant benefits for patients already on antiplatelet therapy. However, patients who concurrently use antiplatelet and anticoagulant therapies may be at a higher risk for chronic subdural hematoma recurrence (39). Conversely, studies have shown that ICU patients who underwent antiplatelet therapy before surgery might achieve better outcomes, possibly because platelet therapy is linked to better initial conditions (40).

These systemic or localized factors underscore the complexity of CSDH recurrence. Interactions between diverse physiological and pathological conditions necessitate a comprehensive patient management approach. A deeper understanding of these contributing factors and their interactions is vital for effective prediction and prevention of CSDH recurrence.

However, certain limitations must be acknowledged. The retrospective design of our study and the reliance on data from a single medical institution might introduce bias and constrain generalizability. These factors could hinder the applicability of our results to wider populations or distinct healthcare settings, where differences in clinical practices, patient demographics, and institutional policies could influence CSDH recurrence patterns. Future research should prioritize multicenter prospective studies to validate our findings.

## Conclusion

Our investigation underscores the capacity of machine learning, especially the random forest (RF) model enhanced with the border-line synthetic minority over-sampling technique (SMOTE), for predicting postoperative chronic subdural hematoma (CSDH) recurrence with remarkable accuracy. Age is the most potent predictor of recurrence, consistent with previous studies. Other significant contributors include elevated levels of AST, abnormal TT, larger HWD, UN, DB, SBP, and history of heart diseases and diabetes. The combined application of LASSO regression, SMOTE, and SHAP analysis enhances the RF model’s precision and interpretability, offering valuable insights for optimizing therapeutic strategies and implementing preventive interventions for high-risk patients. However, our study has limitations, including its retrospective design and reliance on data from a single medical institution. Multicenter prospective studies with diverse populations and different medical contexts are needed to validate our findings.

## Transparency, rigor, and reproducibility summary

Our study utilized data from 447 CSDH patients treated with consecutive burr-hole irrigations at the First Affiliated Hospital of Wenzhou Medical University between December 2014 and April 2019. The Ethics Committee of this institution granted the research ethical approval. Due to the study’s retrospective design, informed consent from participants was considered unnecessary. Upon a valid inquiry, the corresponding author will make available the data used or examined in this research. Of the patients, 312 were designated as the development cohort and 135 as the test cohort. The models incorporated demographic, laboratory, and radiological parameters. The border-line synthetic minority over-sampling technique (SMOTE) addressed data imbalance, while the LASSO regression method identified salient features associated with CSDH recurrence. We employed eight machine learning algorithms to predict hematoma recurrence. Model construction was executed via the XSmartAnalysis website (https://www.xsmartanalysis.com/) using R statistical 3.6.3 and Python 3.7. Evaluation metrics included AUROC, sensitivity, specificity, F1 score, calibration plots, and decision curve analysis (DCA). The RF model displayed exceptional accuracy. Shapley additive explanation (SHAP) analysis enhanced model visualization and interpretability, verifying results and highlighting critical clinical predictors. The rigorous methodology, complemented by diverse machine learning techniques, supports the replicability of our models in comparable clinical contexts. The RF model, with its exemplary performance, stands as a robust tool for predicting postoperative CSDH recurrence, offering valuable insights for therapeutic decision-making and preventive strategies for high-risk patients.

## Data availability statement

The data analyzed in this study is subject to the following licenses/restrictions: the corresponding author can provide the data utilized and/or examined in the present investigation upon a reasonable inquiry. Requests to access these datasets should be directed to nizhihui@wmu.edu.cn.

## Ethics statement

The studies involving humans were approved by Ethics Committee in Clinical Research (ECCR) of the First Affiliated Hospital of Wenzhou Medical University. The studies were conducted in accordance with the local legislation and institutional requirements. Written informed consent for participation was not required from the participants or the participants’ legal guardians/next of kin in accordance with the national legislation and institutional requirements.

## Author contributions

ZN: Conceptualization, Visualization, Writing – original draft. YeZ: Data curation, Formal Analysis, Writing – review & editing. YQ: Software, Validation, Writing – review & editing. XL: Data curation, Writing – review & editing. ZX: Data curation, Writing – review & editing. YiZ: Data curation, Writing – review & editing. YC: Data curation, Writing – review & editing. LH: Writing – review & editing. JY: Supervision, Writing – review & editing. QZ: Supervision, Writing – review & editing.

## Footnotes

^1^ https://www.xsmartanalysis.com/model/list/predict/model/html?mid=6694&symbol=91xd69jB18oL581991bw
